# Cluster randomised control trial protocol for estimating the effectiveness and cost-effectiveness of a complex intervention to increase care home staff influenza vaccination rates compared to usual practice (FLUCARE)

**DOI:** 10.1186/s13063-022-06925-2

**Published:** 2022-12-09

**Authors:** Amrish Patel, Erika Sims, Jeanette Blacklock, Linda Birt, Veronica Bion, Allan Clark, Alys Griffiths, Cecile Guillard, Amber Hammond, Richard Holland, Andy Jones, Liz Jones, Thando Katangwe-Chigamba, Jennifer Pitcher, Po Ruby, Sion Scott, Adam P. Wagner, Saiqa Ahmed, Wasim Baqir, Luke Cook, Tony Dean, David Wright

**Affiliations:** 1 School of Economics, University of East Anglia, Norwich, UK; 2 Norwich Clinical Trials Unit, University of East Anglia, Norwich, UK; 3 School of Healthcare, University of Leicester, Leicester, UK; 4 LOROS Associate Professor in Palliative Care and Frailty, University of Leicester, Leicester, UK; 5 School of Medicine, University of East Anglia, Norwich, UK; 6 Institute of Population Health, University of Liverpool, Liverpool, UK; 7 Leicester Medical School, University of Leicester, Leicester, UK; 8 National Institute for Health Research (NIHR) Applied Research Collaboration (ARC) East of England (EoE), Cambridge, UK; 9 Patient and Public Involvement Representative, NIHR Applied Research Collaboration North West Coast (ARC NWC), Liverpool, UK; 10 Pharmacy Integration Programme, NHS England and Improvement, London, UK; 11 Askham Village Community, Doddington, UK; 12 Norfolk Local Pharmaceutical Committee, Great Bircham, UK

**Keywords:** Residential homes, Nursing homes, Care homes, Long-term care facilities, Influenza vaccination, Staff, Employees, Randomised controlled trial

## Abstract

The care home staff influenza vaccination rate in England is significantly lower than the 75% World Health Organisation recommendation. This represents a substantial potential for resident harm. Barriers to staff vaccination stem from individual and organisational levels. Existing interventions address some but not all barriers and are not underpinned by behavioural science theory. This study aims to estimate the effectiveness and cost-effectiveness of a theory-informed intervention to improve care home staff vaccination rates compared to routine practice.

Set in care homes with both nursing and residential focus, and a range of ownership status, only homes providing long stay care to older people with a staff vaccination rate below 40% are eligible to participate. Participation expressions of interest will be sought using a variety of approaches prior to seeking consent.

The primary outcome measure is the proportion of staff vaccinated at 6 months, with secondary outcome measures being proportion vaccinated at 3 months, numbers of staff sick days, general practitioner and nurse visits to care home, care home resident hospitalisations and mortality.

Based on the assumptions that the mean cluster (care home) size is 54 staff, a coefficient of variation of 0.48, control vaccination rate is 55%, intervention 75%, intra-cluster correlation coefficient of 0.2 and with 90% power, and 20% attrition, we require 39 care homes per arm.

Blocked randomisation will be at the level of care home, stratified by the proportion of non-white care home staff, and implemented by Norwich Clinical Trials Unit.

The intervention comprises co-designed information videos and posters, provision of in-house staff vaccination clinics, and incentive scheme and monthly data collection on trial outcomes. Beyond usual practice, the control arm will additionally contribute monthly data.

Data will be collected at the start, monthly and at 6 months, and analysis will be blind to allocation. Statistical analysis will use the intention-to-treat principle with the difference in vaccination rates between groups compared using a random effect logistic regression model at the staff-level.

This will be the first study to use a theory-informed intervention designed to comprehensively address identified barriers to care home staff influenza vaccination.

**Trial registration:** ISRCTN ISRCTN22729870. Registered on 24 August 22. Secondary identifiers: R209939, IRAS 316820, CPMS 53812.

## Background

In 2020/2021, care home staff flu vaccination levels were reported at 30% in England [1]. This was against a backdrop of the World Health Organisation (WHO) recommending at least 75% of health and social care staff are vaccinated against influenza [2]. There is a direct relationship between resident health outcomes and staff influenza vaccination rates [3, 4], with evidence that both staff health and resident care are improved given higher staff vaccination rates [5, 6]. There is therefore an urgent need for an intervention to improve influenza vaccination rates in care home staff.

The WHO’s 3Cs model of vaccine hesitancy [7] identifies the main barriers of vaccine uptake as convenience, complacency and confidence. Providing vaccination in-house for care home staff directly addresses barriers of convenience and is considered one of the most effective methods for enhancing uptake [8]. A recent UK-based study reported a 30% increase in vaccination rate for care home staff, from 10 to 40%, through the provision of in-house clinics [9]. Nevertheless, whilst confirming the potential benefit of a convenience-based approach to vaccine hesitancy, this study suggests that providing in-house clinics in isolation is unlikely to be sufficient to meet the WHO target of 75% of staff vaccinated.

Between 23 and 67% of care home staff perceive no need for the vaccine as they are healthy [8, 10, 11] (Complacency), and 34 to 60% of care home staff believe that vaccines are either ineffective or cause disease [8, 11–13] (Confidence). The negative influence of peers is well documented [8] and is also understood to be a barrier to vaccine uptake [10]. Consequently, most interventions targeting staff flu vaccine uptake include an information provision component to address barriers of complacency and confidence. Again, whilst information alone is rarely effective [14–16], if tailored to address the identified barriers, the effectiveness of the intervention can be enhanced [14, 17].

Despite the recognition that multi-component interventions are needed to increase vaccine uptake [7, 18], the recommended use of behavioural science theory to develop them [19] has not been reported [20]. To address individual-level barriers, it is therefore necessary to provide access to vaccines in a convenient manner and address individual complacency and confidence regarding vaccination use through the provision of appropriately tailored information.

We therefore developed our intervention by first mapping the known individual-level barriers to the Theoretical Domains Framework (TDF), which is a synthesis of several behavioural science theories [21]. Using an evidence-based approach [22], we identified 31 potentially appropriate behaviour change techniques (BCTs), the active ingredients of interventions, which could potentially form part of the final intervention. A Nominal Group Technique stakeholder consensus group [23] of care home staff and organisational representatives selected those BCTs which met the APEASE criteria (affordable, practicality, effectiveness, acceptability, side-effects, equity) [24] and described how they could be operationalised in practice. This formed the basis of our intervention [25].

In addition to addressing individual-level barriers, it is widely recognised that for staff to undertake a behaviour, they must feel it aligns with the priorities of their organisation [26]. Employer encouragement is a known enabler of staff vaccination uptake [18, 27, 28]. Although there is government-issued guidance for employers, a lack of employer encouragement [18, 29, 30] and direct institutional involvement means that implementation of this guidance is variable [8]. Evidence suggests that incentivisation, performance monitoring and feedback are effective at facilitating managerial support for staff vaccination [31]. Whilst financial inducements are frequently used in the UK to improve the quality of healthcare [32], there is no evidence reporting their effectiveness in care homes to encourage staff vaccination.

**Fig. 1:**
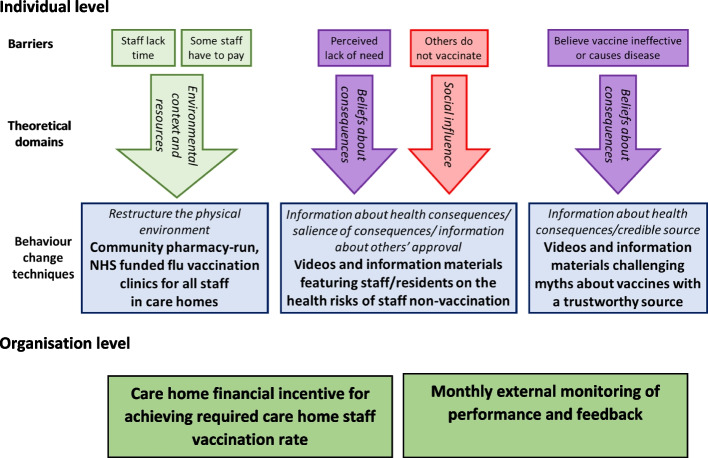
Proposed intervention

Addressing both individual and organisational barriers to staff vaccination, the final model for our intervention is described in Fig. 1. No trials registered on the WHO International Clinical Trials Registry Platform [33] exhibit significant overlap with the proposed intervention.

When considering interventions to enhance care home staff vaccination rates, the National Institute for Health and Care Excellence in England found no cost-effectiveness studies [18]. Consequently, any trial of a complex intervention to enhance care home staff vaccination rates should additionally include this element. In line with national guidance on the development and evaluation of complex interventions [34], feasibility tested the intervention and trial design before finalising our definitive trial protocol.

**Fig. 2:**
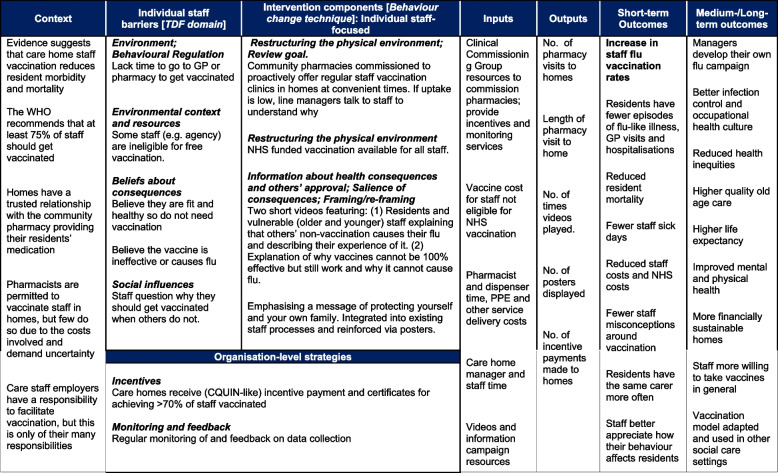
Logic model

Our aim therefore is to assess the effectiveness and cost-effectiveness of an intervention operationalised at both individual and organisational levels to improve care home staff vaccination rates. The overall objectives of this study are to estimate the effect of the intervention on staff vaccination rates (primary outcome) and secondary outcomes identified in the logic model (Fig. 2) and explore the economic impact of the intervention (e.g. cost per vaccination percentage point increase).

Based on our feasibility trial, we propose a two-arm parallel group trial design with a usual care plus monthly data collection control arm. Such a control arm was found to improve data quality without introducing reactivity bias (relatively to other potential control arms) in our feasibility trial.

## Method

### Governance

Norfolk and Waveney Integrated Care system is the Host of FluCare. The University of East Anglia is the trial sponsor and has delegated responsibility for the overall management of the FluCare trial to the Co-Chief Investigators (AP & DW) and Norwich Clinical Trials Unit (NCTU). The project has been overseen by the FluCare Project Steering Committee and the FluCare Data Management and Ethics Committee. The Data Management and Ethics committee is independent of the research team and respects the National Institute of Health Research’s guidelines. Whilst this is a low-risk trial, harms will be reported to and managed by these committees.

### Management

A Project Management Group (PMG), consisting of all co-applicants, meets formally on a quarterly basis to oversee project delivery. A Patient and Public Involvement Group of individuals with personal experience of care homes has been set up, led by AG, which is represented on the PMG, and is actively involved in all project elements. An Expert Advisory Group, consisting of a range of care home experts at local and national levels, was also convened which provides practical and informed advice on intervention design and project delivery and support to problems as they arise.

### Trial design

This is a low-risk, two-arm, open-label, definitive effectiveness and cost-effectiveness trial of FluCare, a behaviour change intervention designed to improve uptake of influenza vaccination by staff in care homes in England, compared to usual care, with an embedded process evaluation (protocol for latter to be published elsewhere).

The design of the trial was informed by a five-arm feasibility trial conducted in 10 care homes. Homes that took part in the feasibility trial are not eligible to take part in the FluCare definitive trial. The feasibility trial confirmed that the usual care (Arm A) and intervention (Arm B) arms in this study will include monthly data collection and the intervention (Arm B) will also include videos, posters and leaflets, in-house vaccination clinics and a financial incentive for care homes.

### Study setting

Community-based private, charity, corporate or local authority residential and nursing homes registered in England to provide care for older adults (aged 65 and over) or dementia care.

### Recruitment

Due to the limited time between feasibility study, protocol redesign and the next flu season, expressions of interest for care home participation were initially sought between May and August 2022. Following expression of interest, community pharmacies or medical practices, local to each care home, willing to provide in-house vaccination clinics were identified.

Five approaches to obtaining expressions of interest will be used:

1. Email will be made with all care homes in England with a staff flu vaccination rate in the 2021/2022 flu season of <40% as identified from the Department of Health and Social Care (DHSC) Capacity Tracker.
2. Publicity materials will be placed in multiple care sector e-newsletters and e-bulletins and via the social media accounts of major care associations (e.g. Care England). The following will be approached to distribute publicity across care homes in England: The Care Quality Commission, The National Care Forum, Pharmacy chains including Boots UK and Day Lewis Pharmacy, local authorities, care home chains and care home network organisations (e.g. CHAIN).
3. Members of the trial team will speak at care home sector associations’ and care home managers’ meetings held online or face-to-face to publicise the study. This includes National Care Forum’s managers’ meetings and Care England’s regional manager and care home manager meetings.
4. Local Clinical Research Networks will circulate the project information to care homes within their region, including those within and external to the Enabling Research in Care Homes (ENRICH) Network. Members of the research team will also present at CRN meetings to care home managers and staff.
5. A social media campaign (using Twitter, LinkedIn, Facebook, and WhatsApp) will be developed and launched.

### Inclusion/exclusion criteria

Care homes expressing interest in participation will be recruited according to the following criteria.

#### Inclusion criteria

- Registration for long stay for adults aged 65 or over, or people with dementia of any age
- Self-reported staff vaccination rate <40% in flu season 2021/2022
- Signed up to, or willing to sign up to the DHSC Capacity Tracker and willing to provide monthly updates on flu vaccine status of staff
- Community pharmacy or medical practice identified as able and willing to deliver in-house vaccination clinics to the participating care home

#### Exclusion criteria

- Fewer than 10 staff members employed by the care home
- Participated in FluCare feasibility study
- Participating in existing trial of behaviour change interventions
- Care home located outside of England

#### Care home staff

##### Inclusion criteria

- All staff (permanent, agency, voluntary) working at the care home at any time from randomisation to end of follow-up will be invited to consent to questionnaire completion. Care homes will also provide anonymised individual-level data (such as details of role, type, vaccination status and sickness) for all staff.

#### Care home residents

##### Inclusion criteria

- Aggregate, non-identifying information, will be provided by care homes on resident mortality and use of health resources (e.g. primary and secondary care). Aggregates will consider all residents irrespective of whether they are permanent or respite residents.

### Pharmacy/GP practice vaccination provider

#### Selection criteria

Following consent, care homes will provide information on their GP practice and/or community pharmacy provider(s) to establish willingness to provide a vaccination service for the purpose of the trial, including out-of-hours provision. The decision of which provider to initially contact will be informed by care home preference.

#### Inclusion criteria

- Willing to provide flu vaccinations within the care home through delivery of up to four vaccination clinics, to care home staff (permanent, agency, voluntary) and residents who were not vaccinated under the usual arrangements.
- Have adequate staff available to provide a flu vaccination service within the care home, including out-of-hours and for new starters.

#### Exclusion criteria

- Unwilling to retain a small number of vaccinations for the purposes of care home staff new starters appearing during the intervention period. Number required to be advised by the related home.

### Remuneration

Participating care homes will receive up to £500 for costs associated with facilitating the research and data collection. Specifically, a care home will receive £200 for all tasks up to and including providing consent to participate in the trial. A further £300 is available for completion of various tasks during the trial (e.g. returning anonymised staff data logs).

A remuneration model for vaccination providers was devised to ensure that for the first eight vaccinations at each clinic, all fixed and variable costs will be appropriately addressed to ensure that the provider will be sufficiently incentivised to offer the service. All additional vaccinations delivered after the first eight will be remunerated as per national funding agreement.

### Allocation and blinding

#### Sequence generation

The allocation sequence will be generated using REDCap, a secure web platform for building and managing online databases and surveys. The allocation sequence will be based on stratified randomisation with strata depending on the ethnic mix of care home staff (reported by care home managers vis the Site Profile Questionnaires (SPQs) prior to randomisation). Blocked randomisation will be undertaken to reduce the risk of imbalance, with a small block size specified given the relatively small number of homes to be randomised.

#### Allocation concealment mechanism

The recruitment and care home facing team will have no access to the allocation sequence.

#### Blinding

Due to the design of the trial, it is not possible to blind the operational and data management members of the research team. Statistics and Health Economics team members will be blinded to the randomisation for the purpose of analysis.

#### Implementation

The NCTU will implement the randomisation process.

### Intervention and usual care

The multi-component intervention (Fig. 1) will comprise of:

- An online video of stakeholders (General Practitioner (GP), pharmacist, care home manager, residents, and care home staff) and supporting information materials (including posters and leaflets)
- Care home incentive scheme comprising of an £850 incentive payment if more than 70% of care home staff receive a flu vaccination as reported on the Department of Health and Social Care Capacity Tracker and in care home staff log. This is in addition to the up to £500 payment for facilitating the research and data collection.
- Monthly monitoring of care home performance (alongside data collection similar to that within the usual care arm)
- GP and/or pharmacy vaccination provision comprising of up to four vaccination clinics organised around care home shifts

Usual care will include monthly data collection. The care home manager/owner will be aware that the care home is participating in the trial, but no additional information will be provided to staff. Outcome data will be requested by the research team on a monthly basis to confirm data quality with feedback provided to the care home manager should issues be identified. The feasibility study suggested no reactivity bias from monthly data collection in isolation.

#### Concomitant care

Care home staff will be able to access NHS care via their usual GP and/or pharmacy provider. Should a member of staff in the intervention home prefer to receive the flu vaccination via their own GP or local pharmacy provider, this is permitted and will be captured in the care home staff log.

### Protocol treatment discontinuation

#### Care home managers/owners

Care home managers/owners may choose to discontinue trial involvement at any time without penalty or loss of benefits to which they would otherwise be entitled. Although not obliged to give a reason for discontinuing their trial involvement, a reasonable effort should be made to establish this reason, whilst remaining fully respectful of the care home manager/owner’s rights.

Care home managers/owners who discontinue protocol treatment will be encouraged to remain in the trial for the purpose of providing follow-up data. Care homes that withdraw after allocation will not be replaced. All care homes that are withdrawn will be included in the data analysis.

#### Flu vaccination providers (intervention only)

GP practice and pharmacy participation in the trial will be voluntary, although providers will be contracted and remunerated for services provided. Should a provider withdraw consent, an alternative provider will be sought and consented and contracted where possible.

### Participant transfers

We acknowledge that care home staff and residents may move between care homes during the trial. To cover new starters and leavers in staff data, the first and late date of staff employment, respectively, will be recorded. Resident data will include information on all residents living at the care home at any point during the trial period.

### Loss to follow-up

#### Care home

Loss to follow-up over the relatively short duration of the influenza season is unlikely. However, the study has been powered to accept loss of care homes as a result of closure or sale (20% attrition has been included in the sample size). In the event that a care home has been sold to a new provider, attempts will be sought to obtain informed consent from the new owner/manager. Movement of staff into and away from the care home will be captured during the trial (see below).

#### Care home staff

As part of the staff data log, when staff leave the care home, care home managers are requested to ensure that the date staff stopped working at the home and their vaccination status at that time are logged.

**Fig. 3:**
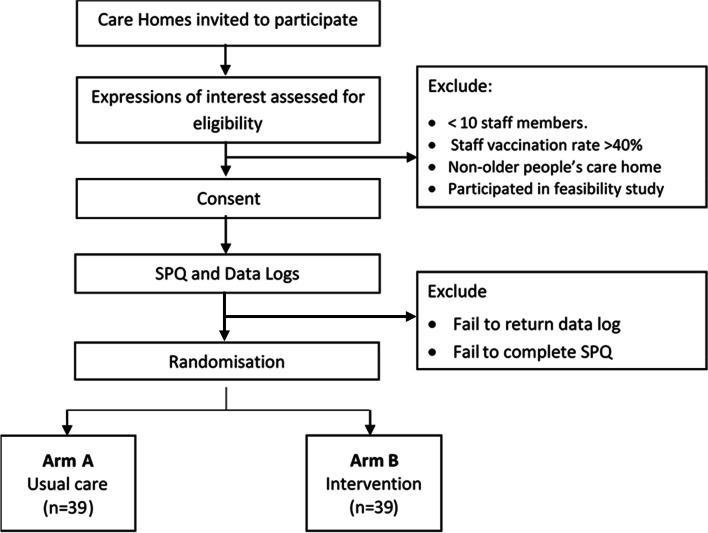
Trial recruitment and randomisation process

A summary of the recruitment and randomisation process is provided in Fig. 3.

### Outcomes

#### Primary outcome

The primary outcome measure is the total number of staff vaccinated in a flu season over the total number of staff employed at any point throughout that flu season. Whilst there is annual variation in the precise dates of flu season in England, flu circulation is highest between October and March each year. We consider all staff (care staff, cleaners, cooks, administrative staff) whether they are directly contracted, bank/agency staff or volunteers.

#### Secondary outcomes

- Staff flu vaccination rate at end of November
- Number of staff sick days
- GP and nurse visits to care home for all residents
- Care home resident hospitalisations
- Care home resident mortality

#### Health economic outcomes

- Intervention delivery and wider health costs (use of primary and secondary costs) compared to impacts on primary and secondary outcomes

### Sample size calculation

Based on the assumptions that mean cluster size is 54 staff, a coefficient of variation of 0.48 (based on a recent study [35]), the control vaccination rate is 55% (assumed higher than the historical rate as COVID has increased interest in vaccination), intervention 75%, intra-cluster correlation coefficient of 0.2 and with 90% power, we require 31 care homes per arm at the two-tailed 5% level of significance (62 total). This would also provide 80% power to detect the same difference in the caregiving (non-caregiving) staff subgroup, assumed 40 per care home (14 per care home). We recruit an additional 8 homes per arm to allow for 20% attrition making the final intended sample size of 78 homes in total. The effect of COVID on the control vaccination rate is uncertain. Our sample size also provides over 90% power to detect a difference between a control rate of 40% and intervention rate of 60%. This would still represent a 50% relative increase in vaccination rates than these homes have achieved historically.

An assumed mean number of 54 staff members is used in the sample size, although it is possible that true number will be larger than this. However, the sample size is relatively unchanged by this and does not reduce to less than 90% until the number of staff members reaches 49. In addition, as the study allows for 20% drop-out of care homes the number of staff members can be reduced even further if the drop-out rate is lower.

### Data collection

Care home managers will return monthly data logs of staff vaccination status, GP and nurse visits to care home sand resident hospitalisations and mortality. In addition, care home managers will be required to complete a Site Profile Questionnaire (SPQ) at the beginning and end of the study to observe contextual or structural changes that may affect implementation during the trial period. The SPQ includes:

- Care home ownership (private/not-for-profit/local authority);
- Size (number of beds and current number of residents);
- Nursing support (registration with/without nursing);
- Staffing (number, role of staff, and contracted shift patterns);
- Policies and procedures: infection control policies, vaccination administration protocols/procedures, vaccination guidance/education provided;
- Pathways available and accessible to staff obtaining flu vaccine (e.g. in care home or independently); and
- Any other structural or staffing changes which may affect implementation during trial period.

### Trial closure

The end of the trial is defined as 1 month following the last interview (within the process evaluation) and return of last data collection form, whichever is the latter, to allow for data entry and data cleaning activities to be completed.

**Fig. 4:**
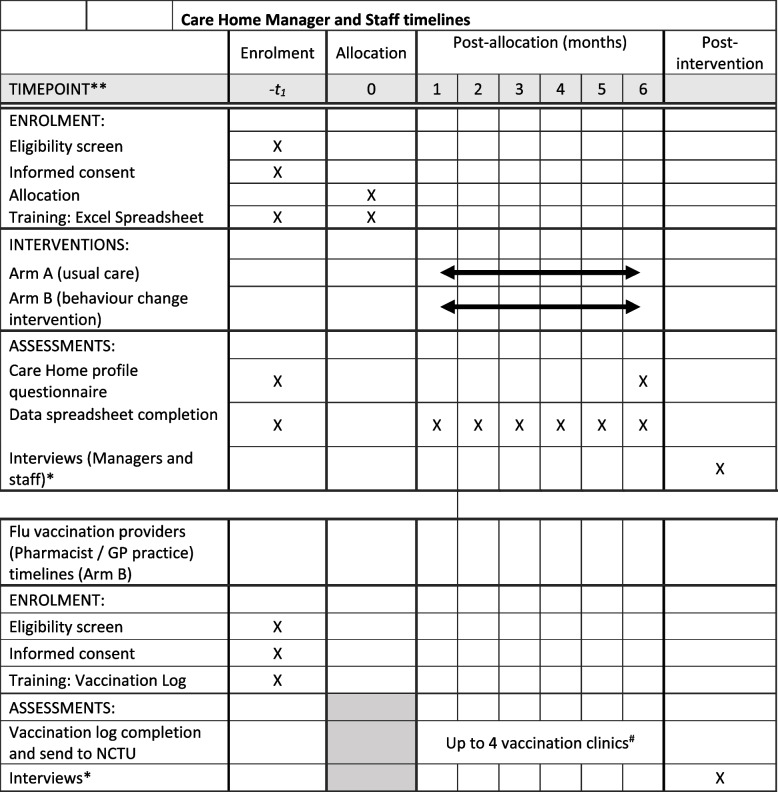
Care home manager and staff timeline

A participant timeline is provided in Fig. 4.

### Data management

Data will be entered under the care home number and participant ID number onto the central database stored on the servers based at NCTU. Access to the database will be via unique, individually assigned (i.e., not generic) usernames and passwords, and only accessible to members of the FluCare trial team, and external regulators if requested. The servers are protected by firewalls and are patched and maintained according to best practice.

Participant identifiable data will be held within the REDCap database separated from the research data by logical separation. Identifiable data will be deleted at the end of the study.

### Data analysis

#### Statistical methods

Analysis based on the intention-to-treat principle, using all available data. The difference in vaccination rates will be presented for each group separately and compared using a random effect logistic regression model at staff level. The random effect will be care home. If staff data are missing, then the results’ sensitivity will be assessed by imputation with two strategies: missing data will be assumed to be not vaccinated; multiple imputation will be attempted using iteratively chained equations. Given the amount of data to base the imputation model on, the primary analysis will remain the observed data analysis. Secondary outcomes will also be compared using random effect models. Assumptions will be checked and if violated then either a nonparametric bootstrap or cluster-summary approach will be used. The analysis will consider firstly, all staff; then all care-giving and non-care-giving staff groups separately. A subgroup analysis will investigate if there is a differential effect in the ethnic minorities staff group. We shall also examine for subgroup effect from selected characteristics of the care home using SPQ responses. Full details will be agreed and documented in the Statistical Analysis Plan (SAP) before the final analysis. Where there is a discrepancy between the SAP and protocol, the SAP will have priority.

#### Health economic methods

We will conduct a within-trial cost-consequences analysis (CCA) comparing costs and outcomes between trial arms across different perspectives/stakeholders (e.g. care homes, NHS and staff). CCA is a standard evaluation approach recognised as being particularly useful for evaluating interventions that have impacts on multiple domains of outcome and perspectives [36, 37].

We will determine the resources involved in, and associated costs of, delivering the FluCare intervention. Resources required for intervention delivery are expected to consist primarily of clinician time to deliver the FluCare clinics and the vaccinations. Information on these and other resources will be collected from clinic logs, process evaluation and augmented with expert opinion as need. Resources will be costed in the most recent cost year for which published NHS and PSS unit costs are available [38].

If the intervention is effective, we will determine the cost per increased percentage point of vaccination rate. We will also consider impacts on care home staff (staff sickness and agency staff utilisation) and resident health (rates of GP visits, nurse visits hospitalisation, and all-cause mortality). Secondary analysis will disaggregate appropriate results by staff type (e.g. care home versus agency employed) and role. We will explore if the intervention costs may be offset by reduction in the use of other resources (e.g. fewer resident GP and nurse visits). Additionally, we will also explore crude valuations of life years gained (for example, noting any differences in resident mortality multiplied by typical survival periods).

The analysis will adopt a ‘within trial’ approach, i.e. up to 6 months of the trial. Given the duration of less than a year, discounting will not be required. In line with the statistical analysis, we will analyse patterns of missing data, and where appropriate, multiple imputation will be used to impute data. Decisions relating to the treatment of missing data will be made in consultation with the study CIs and statisticians. If data is imputed, then results will be presented for both the imputed data as well as a complete case analysis (CCA). Data will be analysed on an intention-to-treat basis. If adjustment for other factors is needed (e.g., care home size), costs and effects will be analysed using appropriate regression-based methods. Analyses will be performed in a variety of packages, likely to include: MS Excel; R; and STATA.

In accordance with NCTU practice, we will draft a health economic analysis plan (HEAP) prior to conducting the economic analysis. This will be shared and discussed with members of the Trial Management Group and other key personnel before analysis is undertaken. Where there is a discrepancy between the HEAP and protocol, the HEAP will have priority.

## Discussion

We believe this to be the first study to develop a care home staff vaccination intervention using behavioural science, and as such has been designed to robustly address barriers at both individual and organisational levels.

Having conducted a feasibility study we are confident of our optimised study design and approaches for data collection. We had assumed that regular data collection in usual-care control arm homes may affect managerial behaviour and cause an increase in staff vaccination rates (reactivity bias); however, this was not proven to be the case when tested. Study data collection may be perceived by care homes as performance monitoring, but according to our outcome measures, this seems to have had no impact when collected in isolation. This could reflect a general lack of vaccination communication and care prioritisation in our sample, which specifically targets care homes with low staff vaccination rates.

Within our feasibility study we identified potential barriers in care home recruitment caused by low care home capacity, and consequently, report a wide range of approaches to improve this. It will be interesting to identify which approaches are most effective as this knowledge will be useful for individuals operating in this context in the future.

The study has been powered on the assumption that there will be some increase in staff vaccination rates in the control arm from previous low levels and such that we have sufficient power to detect a reasonably large change in vaccination rate as a result of our multi-layered intervention. It could be argued that we should have powered our study on a 40% change in vaccination rate such that we demonstrate that the intervention care homes achieve the WHO target i.e., from less than 40% to more than 75%. However, we are firstly comparing the difference in change between the two arms, which may be less, but still sufficient to achieve 75% in the intervention arm. Secondly, the number of homes required to detect such a potentially large difference would be very small and we may then not detect a significant but meaningful difference of 20%.

The planned health economic component will be a CCA, rather than the more common cost-utility analysis (CUA), in which cost impacts are compared to impacts measured in terms of quality-adjusted life years (QALYs). In the UK, CUAs typically adopt the perspective of the national health service and personal social services. Here, CCA is more suited to this evaluation context where the intervention impacts on multiple domains and perspectives [36, 37]. Furthermore, to ensure acceptability and feasibility of trial delivery in this setting, primary data collection — such as asking staff or residents to complete measures that would allow calculation of QALYs — needed to be kept at a minimum. The proposed analysis draws on data that can be provided by a few key staff at each home, reducing the trial impact in this already resource-stretched setting.

We are also confident that data collection is both feasible and practical, only requiring care home managers to complete the Site Profile Questionnaire at the start and end of the trial, and to provide monthly data only on number of staff, staff leavers and starters, plus vaccination status and aggregate resident data. In our feasibility trial, the remuneration offered for participation was found to be sufficient to encourage data provision. Similarly, the financial model for encouraging a vaccine provider to engage with in-house vaccination clinic provision was shown to be sufficient.

Active involvement of our Expert Advisory Group, which consists of a range of healthcare professionals with expertise in care homes, individuals from a range of national care home representative organisations plus experts in Social Care operating at the government level was central to the design of our final research protocol. Our patient and public involvement members have been involved in all stages of the project, from inception to providing advice on the different elements of the intervention e.g., poster and video, providing guidance on how to enhance our approach to recruitment and how to communicate effectively with care homes and in the qualitative analysis of the data from our process evaluation. This regular input provides greater confidence in the potential for our intervention to be effective and in the delivery of our research project.

The trial results will be published in an open access, peer-reviewed journal and we will work with our public and patient involvement group and expert advisory panel to develop a dissemination strategy appropriate to reaching relevant target audiences. All members of the FluCare team that have contributed to the protocol design and written or commented on the paper will be eligible for authorship of outputs.

### Trial status

Protocol version 1.1, 5 August 2022. Recruitment started on 31 August 2022. Recruitment is planned to be completed by 30 November 2022

For significant protocol modifications, an ethics amendment will be sought and the updated protocol version sent to all relevant parties (registries, funder etc).

## Data Availability

Requests for access to the FluCare trial dataset should be made to Dr Amrish Patel (Amrish.Patel@uea.ac.uk) and/or Researchsponsor@uea.ac.uk. During the trial requests will be reviewed by the Chief Investigators, Trial Management Group and the Trial Steering Committee. Post-trial requests will be reviewed by the Chief Investigators (and representatives from the Trial Management Group where available) and Sponsor. Requests will be reviewed to ensure that there is no conflict with the objectives of the FluCare trial (pre-publication of FluCare findings) or funder constraints. All data requests will be subject to a data-sharing agreement with the University of East Anglia.

## Abbreviations

CCA: Cost-consequences analysis
CUA: Cost-utility analysis
GP: General Practitioner
NCTU: Norwich Clinical Trials Unit
PMG: Project Management Group
QALY: Quality Adjusted Life Year
SPQ: Site Profile Questionnaire
WHO: World Health Organisation
